# Esquirol on Insanity

**Published:** 1845-06

**Authors:** 


### ESQUIROL ON INSANITY.

The American profession is indebted to Dr. E. K. Hunt for a translation of this standard work, which was issued, a few weeks since, by Lea & Blanchard in their accustomed style of accuracy and neatness. Esquirol is a name so familiar to all who have paid the slightest attention to maladies of the mind, that it is quite unnecessary to say anything in commendation of any publication from his pen. He stands before the world as a master in his art. His authority is the highest on all the questions to which he devoted the energies of his active life. His treatise has become a classic in our profession, and we cordially hail this translation which will place it in the hands of so many of our brethren. Y.

